# Facing the Challenges of Developing Fair Risk Scoring Models

**DOI:** 10.3389/frai.2021.681915

**Published:** 2021-10-14

**Authors:** Gero Szepannek, Karsten Lübke

**Affiliations:** ^1^ Institute of Applied Computer Science, Stralsund University of Applied Sciences, Stralsund, Germany; ^2^ Institute for Empirical Research and Statistics, FOM University of Applied Sciences, Dortmund, Germany

**Keywords:** scoring, machine learning, causal inference, German credit data, algorithm fairness, explainable machine learning

## Abstract

Algorithmic scoring methods are widely used in the finance industry for several decades in order to prevent risk and to automate and optimize decisions. Regulatory requirements as given by the Basel Committee on Banking Supervision (BCBS) or the EU data protection regulations have led to an increasing interest and research activity on understanding black box machine learning models by means of explainable machine learning. Even though this is a step into a right direction, such methods are not able to guarantee for a fair scoring as machine learning models are not necessarily unbiased and may discriminate with respect to certain subpopulations such as a particular race, gender, or sexual orientation—even if the variable itself is not used for modeling. This is also true for white box methods like logistic regression. In this study, a framework is presented that allows analyzing and developing models with regard to fairness. The proposed methodology is based on techniques of causal inference and some of the methods can be linked to methods from explainable machine learning. A definition of counterfactual fairness is given together with an algorithm that results in a fair scoring model. The concepts are illustrated by means of a transparent simulation and a popular real-world example, the German Credit data using traditional scorecard models based on logistic regression and weight of evidence variable pre-transform. In contrast to previous studies in the field for our study, a corrected version of the data is presented and used. With the help of the simulation, the trade-off between fairness and predictive accuracy is analyzed. The results indicate that it is possible to remove unfairness without a strong performance decrease unless the correlation of the discriminative attributes on the other predictor variables in the model is not too strong. In addition, the challenge in explaining the resulting scoring model and the associated fairness implications to users is discussed.

## 1 Introduction

The use of algorithmic scoring methods is very common in the finance industry for several decades in order to prevent risk and to automate and optimize decisions (Crook et al., 2007). Regulatory requirements as given by the Basel Committee on Banking Supervision (BCBS) (European Banking Authority, 2017) or the EU data protection regulations (Goodman and Flaxman, 2017) have led to an increasing interest and research activity on understanding black box machine learning models by means of explainable machine learning (cf. e.g., Bücker et al., 2021). Even though this is a step into a right direction, such methods are not able to guarantee for a fair scoring as machine learning models are not necessarily unbiased and may discriminate with respect to certain subpopulations such as a particular race, gender, or sexual orientation—even if the variable itself is not used for modeling. This is also true for white box methods like logistic regression.

In the study by O’Neil (2016), several popular examples are listed as to how algorithmic decisions enter and potentially negatively impact everyday lives. An expert group on the AI setup by the European Commission has worked out an assessment list for trustworthy artificial intelligence (ALTAI), where one requirement consists in diversity, non-discrimination, and fairness (EU Expert Group on AI, 2019).

There are different definitions of algorithm fairness. An overview is given by Verma and Rubin (2018) and will be summarized in Section 2. In the remainder of the section, the framework of counterfactual fairness is introduced as well as an algorithm that allows developing fair models based on techniques of causal inference (Pearl et al., 2016). In the study by Kusner and Loftus (2020), three tests on algorithmic fairness are presented.

Subsequently, in Section 3, fairness is discussed from the usage context of risk scoring models: as opposed to existing crisp fairness definitions a group unfairness index is introduced to quantify the degree of fairness of a given model. This allows for a fairness comparison of different models. Furthermore, it is shown how partial dependence profiles (Friedman, 2001) as they are popular in the field of explainable AI can be adapted in order to enable a visual fairness analysis of a model.

With the scope of the financial application context the aforementioned algorithm is applied to real-world data of credit risk scoring: the German Credit data which is publicly available by the UCI machine learning data repository (Dua and Graff, 2017). The data are very popular and have been used in numerous studies (cf. e.g., Louzada et al., 2016). In contrast to past publications, we used a corrected version of the data in our study as it has turned out that the original data were erroneous (Groemping, 2019). The latter observation has to be highlighted as the data from the UCI repository have been frequently used in credit scoring research during the last decades and thus have strongly influenced research results during the last years. The data and its correction are described in Section 4.1. In Section 4.2, the design of a simulation study based on the corrected German credit data is set up and its results are presented in Section 5: both a traditional scorecard model using weights of evidence and logistic regression as well as a fairness-corrected version of it are compared on the simulated data with regard to the trade-off between fairness and predictive accuracy. Finally, a summary of our results is presented in Section 6.

## 2 Fairness Definitions

### 2.1 Overview

In the literature, different attempts have been made in order to define fairness. An overview together with a discussion is given in Verma and Rubin (2018). In this section, a brief summary of important concepts is given using the following notation:

• *Y* is the observed outcome of an individual. Credit risk scoring typically consists in binary classification, that is, *Y* = 1 denotes a good and *Y* = 0 denotes a bad performance of a credit.
• *P* is a set of one or more protected attributes. With regard to these attributes fairness should be ensured.
• *X* are the remaining attributes used for the model (*X* ∩ *P* = ∅).
• *S* is the risk score, typically a strictly monotonic function in the posterior probability *Pr*(*Y* = *y*|*X* = *x*, *P* = *p*). Without loss in generality, in the context of this study both are chosen to be identical.
• $\hat{Y}\in \left\{0,1\right\}$ is a decision based on the score usually given by $\hat{Y}=1_{\left\{S\left(Y=1\right)\geq s_{0}\right\}}$ where *s*_0_ is cut off.

Typical examples of protected attributes are gender, race, or sexual orientation. An intuitive requirement of fairness is as follows: 1) to use only variables of *X* but no variables of *P* for the risk score model (unawareness). Note that while it is unrealistic that an attribute like sexual orientation directly enters the credit application process it has been demonstrated that this information is indirectly available from our digital footprint such as our Facebook profile (Youyou et al., 2015) and recent research in credit risk modeling proposes to extend credit risk modeling by including such alternative data sources (De Cnudde et al., 2019). From this it is easy to see the fairness definition of unawareness is not sufficient.

Many fairness definitions are based on the confusion matrix as it is given in Table 1: Confusion matrices are computed depending on *P* and resulting measures are compared.

**TABLE 1:** Confusion matrix and measures derived from it.

	*Y* = 1	*Y* = 0	
$\hat{Y}=1$	True positives	False positives	Precision: $Pr\left(Y=1|\hat{Y}=1\right)$
$\hat{Y}=0$	False negatives	True negatives	
	Sensitivity: $Pr\left(\hat{Y}=1|Y=1\right)$	Specificity: $Pr\left(\hat{Y}=0|Y=0\right)$	

Fairness definitions related to the acceptance rate $Pr\left(\hat{Y}=y\right)$ are as follows: 2) *Group fairness* $Pr\left(\hat{Y}=1|P\right)=Pr\left(\hat{Y}=1\right)$ requires the acceptance rate to be independent of the protected attributes *P*. In addition, 3) *conditional statistical parity* $Pr\left(\hat{Y}=1|X,P\right)=Pr\left(\hat{Y}=1|X\right)$ requires this independence to hold for any combination of realizations of a set of legal predictors *X*. 4) *Equal opportunity* $Pr\left(\hat{Y}=1|Y=1,P\right)=Pr\left(\hat{Y}=1|Y=1\right)$ and 5) *predictive equality* $Pr\left(\hat{Y}=0|Y=0,P\right)=Pr\left(\hat{Y}=0|Y=0\right)$ are based on the sensitivity and specificity while *balance* for the positive and negative class require the expected scores 6) *E* (*S*|*Y* = 1, *P*) = *E* (*S*|*Y* = 1) and 7) *E* (*S*|*Y* = 0, *P*) = *E* (*S*|*Y* = 0) to be independent of the protected attributes.

Fairness definitions based on the predicted posterior probability *Pr* (*Y*) are as follows: 8) *Predictive parity* $Pr\left(Y=1|\hat{Y}=1,P\right)=Pr\left(Y=1|\hat{Y}=1\right)$ ensures the same precision independent of the protected attributes. In addition, 9) *conditional use accuracy equality* $Pr\left(Y=y|\hat{Y}=y,P\right)=Pr\left(Y=y|\hat{Y}=y\right)$ extends this definition to all levels of *Y*. In contrast, 10) *calibration* *Pr*(*Y* = 1|*S* = *s*, *P*) = *Pr*(*Y* = 1|*S* = *s*) ensures same predicted posterior probabilities given a score, independently of the protected attributes.

An alternative yet intuitive fairness definition is given by 11) *individual fairness*: similar individuals *i* and *j* should be assigned similar scores, independently of the protected attributes: *d*_1_ (*S*(*x*_*i*_), *S* (*x*_*j*_)) ≤ *d*_2_ (*x*_*i*_, *x*_*j*_) where *d*_1_ (.,.) and *d*_2_ (.,.) are distance metrics in the space of the scores and the predictor variables, respectively. 12) *Causal discrimination* requires a credit decision $\hat{Y}\left(x\right)$ for two individuals with identical values in the attributes *X* = *x* to be constant independently of the protected attributes *P*. 13) *Counterfactual fairness* additionally requires that $\hat{Y}$ does not depend on any descendant of *P* and will be explained in detail in Section 2.3. For an in-depth overview and discussion of the different fairness definitions it is referred to in Verma and Rubin (2018).

It should be noted that all these criteria can be incompatible so that it can be impossible to create a model that is fair with respect to all criteria simultaneously (Chouldechova, 2016).

### 2.2 Causal Inference

Pearl (2019) distinguishes three levels of causal inference as follows:

1) *Association*: *Pr* (*y*|*x*): Seeing: “What is?,” that is, the probability of *Y* = *y* given that we observe *X* = *x*.
2) *Intervention*: *Pr* (*y*|*do*(*x*)): Manipulation: “What if?,” that is, the probability of *Y* = *y* given that we intervene and set the value of *X* to *x*.
3) *Counterfactuals*: *Pr* (*y*_*x*_|*x*′, *y*′): Imagining: “What if I had acted differently?,” that is, the probability of *Y* = *y* if *X* had been *x* given that we actually observed *x*′, *y*′.

For levels 2 and 3, subject matter knowledge about the causal mechanism that generates the data is needed. This structural causal model can be encoded in a directed acyclic graph (DAG). The basic elements of such a graph reveal if adjustment for variable *C* may introduce or remove bias in the causal effect of *X* on *Y* (see e.g., Pearl et al., 2016):

• *Chain*: *X* → *C* → *Y*, where *C* is a mediator between *X* and *Y* and adjusting for *C* would mask the causal effect of *X* on *Y*.
• *Fork*: *X* ← *C* → *Y*, where *C* is a common cause of *X* and *Y* and adjusting for *C* would block the noncausal path between *X* and *Y*.
• *Collider*: *X* → *C* ← *Y*, where *C* is a common effect of *X* and *Y* and adjusting for *C* would open a biasing path between *X* and *Y*.

Luebke et al. (2020) provide easy to follow examples to illustrate these. In order to calculate the counterfactual (level 3) the assumed structural causal model and observed data is used in a three-step process as follows:

1) *Abduction*: Use the evidence, that is, data to determine the exogeneous variables, for example, error term, in a given structual causal model. For example assume a causal model with additive exogeneous *Y* = *f*(*X*) + *U* and calculate *u* for a given observation *x*′, *y*′.
2) *Action*: Substitute in the causal model the values for *X* with the counterfactual *x* (instead of *x*′).
3) *Prediction*: Calculate *Y* based on the previous steps.

For a more detailed introduction the reader is referred to Pearl et al. (2016).

### 2.3 Counterfactual Fairness

Causal counterfactual thinking enables the notion of counterfactual fairness as follows:

$$Pr\left({Ŷ}_{p}|X=x,P=p\right)=Pr\left({Ŷ}_{p\prime }|X=x,P=p^{}\right)$$

“Would the credit decision have been the same if the protected attribute had taken a different value (e.g. if the applicant had been male instead of female)?”

Kusner et al. (2017) present a FairLearning algorithm which can be considered as a preprocessing debiaser in the context of Agrawal et al. (2020), that is, a transformation of the attributes before the modeling by means of the subsequent machine learning algorithm. It consists of three levels as follows:

1) Prediction of *Y* is only based on non-descendants of *P*.
2) Use of postulated background variables.
3) Fully deterministic model with latent variables where the error term can be used as an input for the prediction of *Y*.

It should be noted that in general causal modeling is non-parametric and therefore any machine learning method may be employed. In order to illustrate the concept we utilize a (simple) linear model with a least squares regression of the attributes *X* (e.g., status in the example below) on the protected attributes *P* (e.g., gender) assuming an independent error. The resulting residuals (*E*) are subsequently used to model the *Y* (e.g., default) instead of the original attributes *X* which may depend on the protected attributes *P*. Our algorithm can be summarized as follows:

1) Regress X on P.
2) Calculate residuals $E=X-\hat{X}$ .
3) Model *Y* by *E*.

## 3 Analyzing Fairness of Credit Risk Scoring Models

### 3.1 Quantifying Fairness

The definitions as presented in the previous subsection are crisp in the sense that a model can be either fair or not. It might be desirable to quantify the degree of fairness of a model. In Section 2 different competing definitions of fairness are presented. It can be shown that sometimes they are even mutually exclusive, for example, in Chouldechova (2016) it is shown that for a calibrated model (i.e., *Pr*(*Y* = 1|*S* = *s*, *P*) = *Pr*(*Y* = 1|*S* = *s*), cf. above) not both equal opportunity (i.e., $Pr\left(\hat{Y}=1|Y=1,P\right)=Pr\left(\hat{Y}=1|Y=1\right)$ ) and predictive equality (i.e., $Pr\left(\hat{Y}=0|Y=0,P\right)=Pr\left(\hat{Y}=0|Y=0\right)$ ) can be given as long as there are different prior probabilities *Pr*(*Y* = 1|*P* = *p*) with respect to the protected attributes. Although each of the definitions can be motivated for the credit scoring business context the group fairness which takes into account for the acceptance rates seems to be of major relevance. For this reason we concentrate on group fairness in order to quantify fairness of credit scoring models: By $Pr\left(\hat{Y}=1|P\right)$ the distribution of $\hat{Y}$ with regard to the protected attributes are given. If *P* ∈ {0, 1} is binary, like gender, a popular measure from scorecard development can be adapted, the population stability index (PSI, cf. e.g., Szepannek, 2020). Moreover, there are thumb rules available from literature that allows for an interpretation: *PSI* > 0.25 is considered as unstable (Siddiqi, 2006). For our purpose, a group unfairness index (GUI) is defined as follows:

$$GUI=\overset{1}{\underset{\hat{Y}=0}{\sum }}\left(Pr\left(\hat{Y}|P=1\right)-Pr\left(\hat{Y}|P=0\right)\right)\;\log\left(\frac{Pr\left(\hat{Y}|P=1\right)}{Pr\left(\hat{Y}|P=0\right)}\right).$$ (1)

Analogously a similar index can be defined for other fairness definitions based on the acceptance rate $Pr\left(\hat{Y}=y\right)$ such as equal opportunity (cf. Section 2.1). Nonetheless for the purpose of this study and the application context of credit application risk scoring we restrict to group fairness.

### 3.2 Visual Analysis of Fairness

A risk score *S*≔*Pr*(*Y* = *y*|*X* = *x*, *P* = *p*) that is independent of *P* necessarily results in group fairness as $\hat{Y}=1_{\left\{S\left(Y=1\right)\geq s_{0}\right\}}$ . From the field of explainable machine learning partial dependence profile (P*DP)* plots (Friedman, 2001) are known to be one of the most popular model-agnostic approaches for the purpose of understanding feature effects. The idea of PDPs can be adapted to visualize a model’s partial profile with respect to the protected attributes *P* even if they are not necessarily among the predictors *X*:

$$PDP\left(P\right)=\int S\left(Y=1|X,P\right)dF_{X},$$ (2)

that is, average prediction given the protected attributes *P* take the value *p*. For our purpose, for a data set with *n* observations (*x*_*i*_, *p*_*i*_) a protected attribute dependence profile can be estimated by the conditional average

$$\hat{{PDP}_{\;}}\left(p\right)=\frac{1}{n_{p}}\overset{}{\underset{p_{i}=p}{\sum }}S\left(Y=1|p_{i},x_{i}\right).$$ (3)

In case of a fair model the protected attribute dependence profile should be constant.

## 4 Simulation Experiment

### 4.1 From German Credit Data to South German Credit Data

Traditionally, credit scoring research has suffered from a lack of available real-world data for a long time as credit institutes are typically not willing to share their internal data. The German credit data have been collected by the StatLog project (Henery and Taylor, 1992) and go back to Hoffmann (1990). They are freely available from the UCI machine learning repository (Dua and Graff, 2017) and consist of 21 variables: 7 numeric as well as 13 categorical predictors and a binary target variable where the predicted event denotes the default of a loan. The default rate on the data is has been oversampled to 0.3 on the available UCI data while the original sources report a prevalence of bad credits around 0.05.

In the recent past, a few data sets have been made publicly available, for example, by the peer-to-peer lending company LendingClub^1^ or FICO^2^ but a still a huge number of studies rely on the German credit data (Louzada et al., 2016). This is even more notable as in Groemping (2019) it has been figured out that the data available in the UCI machine learning repository are erroneous, for example, the percentage of foreign workers in the UCI data is 0.963 (instead of 0.037) because the labels have been swapped. In total, eleven of the 20 predictor variables had to be corrected. For seven of them (A1: Status of existing checking account, A3: Credit history, A6: Savings account/bonds, A12: Property, A15: Housing, and A20: Foreign worker) the label assignments were wrong and for one of them (A9: Personal status and sex) even two of the levels (female non-singles and male singles) had to be merged as they can’t be distinguished anymore. In addition, four other variables (A8: Installment rate in percentage of disposable income, A11: Present residence since, A16: Number of existing credits at this bank, and A18: Number of people being liable to provide maintenance for) which originally represent numeric attributes that are only available after binning such that their numeric values represent nothing but group indexes. For this reason the values of these variables are replaced by the corresponding bin labels. A table of all the changes can be found in the Supplementary Table S1. The corrected data set has been made publicly available on the UCI machine learning repository under the name south German credit data^3^ (Groemping, 2019).

For further modeling in this study the data have been randomly split into 70% training and 30% test data. Note that the size of the data is pretty small but as traditional scorecard development requires a manual plausibility check of the binning cross validation is not an option here (cf. also Szepannek, 2020).

### 4.2 Simulation of the Protected Attribute

A simulation study is conducted in order to compare both a traditional and a fair scoring model under different degrees of influence of the protected attributes. Note that the original variable personal status and sex (A9) does not allow for a unique distinction between men and women (cf. previous subsection) and cannot be used for this purpose. For this reason this variable has been removed from the data.

In traditional scorecard modeling information values (IVs, Siddiqi, 2006) are often considered to assess the ability of single variables to discriminate good and bad customers. Table 2 shows the IVs for the scorecard variables. In order to analyze the impact of building fair scoring models the data have been extended by an artificial protected variable Gender to mimic A9. For this purpose the variable Status with largest IV has been selected to construct the new protected variable. As it is shown in Table 3, in the first step two of the status-levels are assigned to women and the other two variables are assigned to men, respectively. In consequence, women take the lower risk compared to men from their corresponding status levels in the artificial data. The resulting graph is

Gender→Status→Y.

**TABLE 2:** Information values of the scorecard variables and the removed variable personal status and sex.

Variable	Status	Credit history	Duration	Purpose	Savings
IV	0.672	0.298	0.254	0.238	0.154

**TABLE 3:** Construction of the variable gender for the training data.

Status	Female	Male	Default rate
No checking account	0	197	0.452
… < 0 DM	0	184	0.397
0 < = … < 200 DM	44	0	0.205
… > = 200 DM/salary for at least 1 year	275	0	0.105

Note that the model does not use the ethically critical variables Personal status and sex and Foreign worker nor the new simulated protected variable Gender (cf. Section 5) and thus fulfills the definition of fairness through *unawareness*. But as it can be seen in the results of the next Section simply preventing protected variables from entering the model is not sufficient to obtain a fair scoring model (cf. also Kusner et al., 2017).

As Lemma 1 of Kusner et al. (2017) states $\hat{Y}$ can only be counterfactually fair if it is function of the non-descendants of *P* we illustrate the concept by using a chain where by construction the attribute *X* (Status) is a descendant of the protected attribute *P*. The idea can be generalized for larger sets of *X*, *P*.

In a second step the strength effect of gender on the status is varied by randomly switching between 0*%* and 50*%* of the males into females and vice versa. As a result, the designed effect of gender on status is disturbed to some extent and only holds for the remaining observations. The degree of dependence between both categorical variables is measured using Cramer’s V (Cramér, 1946, 282).

## 5 Results and Discussion

**FIGURE 1:**
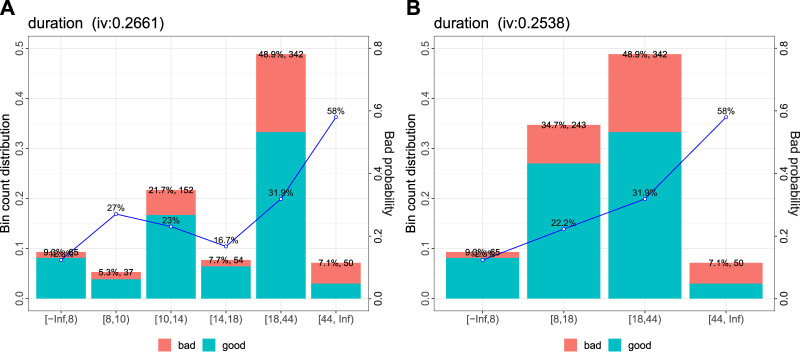
Automatically created bins **(A)** for the variable duration and manual update **(B)**: a plausible trend of increasing risk with increading duration.

Logistic regression still represents the gold standard for credit risk scorecard modeling (Crook et al., 2007; Szepannek, 2020) even if in the recent past many studies have demonstrated potential benefits from using modern machine learning algorithms (cf. e.g., Lessmann et al., 2015; Bischl et al., 2016; Louzada et al., 2016). For this reason, a traditional scorecard using logistic regression is created as a baseline model for the simulation study. The model is built using preliminary automatic binning (based on the *χ* ^2^ statistic and a maximum set to six bins per variable) with subsequent assignment of weights of evidence ( $WOE\left(x\right)=\log\left(\frac{f\left(x|y=1\right)}{f\left(x|y=0\right)}\right)$ ) to the bins (Xie, 2020). For plausibility reasons the bins of four of the variables (Duration, Employment duration, Amount, as well as Age) are manually updated where only one of them (Duration) did enter the final model after BIC based stepward forward variable selection (cf. Figure 1).

For plausibility reasons (i.e., the observed default rates for the different levels) the variable property has been removed from the data as from a business point of view there is no plausible reason for the observed increase in risk for owners of cars, life insurance or real estate (cf. Table 4). After forward variable selection using BIC on the training data the resulting scorecard model uses five input variables as they are listed in Table 2. The equation of the resulting logistic regression model is given in Table 5. The corresponding scorecard model with frequencies and default rates for all classes can be found in Table 6.

**TABLE 4:** Default rates of the variable property on the training data.

Property	Non-default	Default
Unknown/no property	0.79	0.21
Car or other	0.72	0.28
Building soc. savings agr./life insurance	0.73	0.27
Real estate	0.54	0.46

**TABLE 5:** Coefficients of the logistic regression model.

Variable	${\hat{\beta }}_{j}$
Intercept	−0.910
WOE (Status)	0.830
WOE (Duration)	1.063
WOE (Purpose)	1.098
WOE (Credit history)	0.838
WOE (Savings)	0.758

**TABLE 6:** Resulting scorecard model. In practice it is usual to assign scorecard points to the posterior probabilities as given by the score. Here, a calibration with 500 points at odds of 1/19 and 20 points to double the odds is used.

Variable	Bin	Points	Default rate	Distribution	#Good	#Bad
Basepoints	—	441	—	—	—	—
Status	No checking account	–17	0.45	0.28	108	89
Status	… < 0 DM	–12	0.40	0.26	111	73
Status	0 < = … < 200 DM	11	0.20	0.06	35	9
Status	… > = 200 DM/salary for at least 1 year	29	0.11	0.39	246	29
Duration	(−Inf, 8)	32	0.12	0.09	57	8
Duration	(8, 18)	10	0.22	0.35	189	54
Duration	(18, 44)	–5	0.32	0.49	233	109
Duration	(44, Inf)	–38	0.58	0.07	21	29
Purpose	Repairs	–25	0.47	0.05	19	17
Purpose	Domestic appliances, business, others, radio/television	–16	0.40	0.29	124	81
Purpose	Retraining	–2	0.30	0.10	49	21
Purpose	Car (used)	2	0.27	0.17	89	33
Purpose	Furniture/equipment	17	0.19	0.27	151	35
Purpose	Car (new), vacation	23	0.16	0.12	68	13
Credit history	Delay in paying off in the past, critical account/other credits elsewhere	–32	0.60	0.08	22	33
Credit history	No credits taken/all credits paid back duly	–3	0.31	0.54	261	116
Credit history	Existing credits paid back duly till now	0	0.29	0.09	45	18
Credit history	All credits at this bank paid back duly	18	0.16	0.29	172	33
Savings	Unknown/no savings account	–5	0.34	0.60	276	141
Savings	… < 100 DM	–3	0.32	0.11	52	24
Savings	100 < = … < 500 DM	13	0.18	0.06	37	8
Savings	500 < = … < 1000 DM, … > = 1000 DM	15	0.17	0.23	135	27

In addition to the traditional scorecard baseline model fair models are developed according to the algorithm presented in Section 2.3 by regressing *WOE* (*Status*) on the protected attribute Gender and using the residuals instead of the original variable Status as a new input variable, a level 3 assumption for a causal model in Kusner et al. (2017).

**FIGURE 2:**
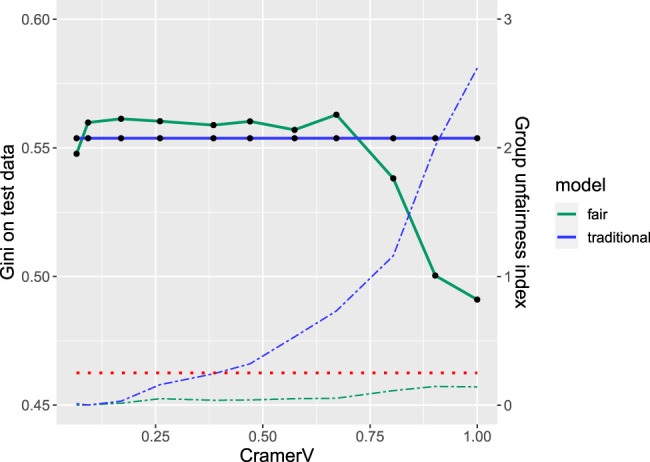
Fairness-performance trade-off: performance (solid) and unfairness (dashed) for traditional (blue) and fairness-corrected (green) model for different levels of correlation between the protected variable gender and the prediction variable status. The red dotted line indicates the thumb rule for unfairness.

Figure 2 shows the results of the simulation study for different levels of dependence (measured by Cramer’s V) between protected variable Gender and Status in terms of both: performance of the model (in terms of the Gini coefficient) as well as the group unfairness index calculated on the training data. Note that for companies depending on the business strategy and corresponding acceptance rates it can be more suitable to put more emphasis on other performance measures such as the partial AUC (Robin et al., 2011) or the expected maximum profit (Verbraken et al., 2014). Nonetheless, for the purpose of this study we decided to use the Gini coefficient $=2\left(AUC-\frac{1}{2}\right)$ as it represents the most commonly used performance measure in credit scoring. In order to compute the GUI a cut off *s*_0_ for the score *S* has to be defined: For the scope of the simulation study within this study the cut off has been set to the portfolio default rate 0.3, that is, an application is rejected if $\hat{P}\left(Y\right)>0.3$ . In practice, rejecting all customers with a risk above average will lead to an unrealistically high rejection rate. Therefore also the GUI is computed for a second cut off value of *s*_0_ = 0.5. Both results are given in Table 7.

**TABLE 7:** Results of the simulation study: Group unfairness index (GUI) of both the traditional as well as the fair model and performance on the test data of the fair model for different levels of dependence (Cramer’s V) between the protected attribute and the variable status.

Cramer’s V	*GUI*_*trad*._(0.3)	*GUI*_*fair*_ (0.3)	*GUI*_*trad*._(0.5)	*GUI*_*fair*_ (0.5)	*Gini*_*fair*, *test*_
1.00	2.620	0.141	1.477	0.078	0.491
0.90	2.014	0.145	1.130	0.120	0.500
0.80	1.160	0.112	0.837	0.057	0.538
0.67	0.732	0.053	0.384	0.005	0.563
0.57	0.530	0.050	0.271	0.002	0.557
0.47	0.321	0.040	0.149	0.001	0.560
0.38	0.243	0.037	0.128	0.000	0.559
0.26	0.158	0.049	0.063	0.003	0.560
0.17	0.030	0.014	0.018	0.003	0.561
0.09	0.000	0.001	0.005	0.001	0.560
0.07	0.011	0.000	0.000	0.015	0.548

The solid lines indicate performance on the test data for the traditional (blue) and the fair model (green). The traditional model is unaffected by the protected variable and thus of constant performance with a Gini coefficient of 0.554. Remarkably, for some of the simulated data sets the fair model even slightly outperforms the traditional one which might be explained by the small number of observations in the data resulting in a large 95*%* (bootstrap) confidence interval (Robin et al., 2011) of (0.437,0.667) and only small performance decrease due to the fairness correction. The corresponding dashed lines show the group unfairness index of both models where the additional dotted red line represents the thumb rule threshold of 0.25 indicating unfairness. For the traditional model the threshold is already exceeded for dependencies as small as Cramer’s V = 0.3 while for the fair model it always stays below the threshold. Even more interesting for small and moderate levels of dependence (Cramer’s V ≤ 0.6) there is no performance decrease observed while at the same time fairness can be increased.

**FIGURE 3:**
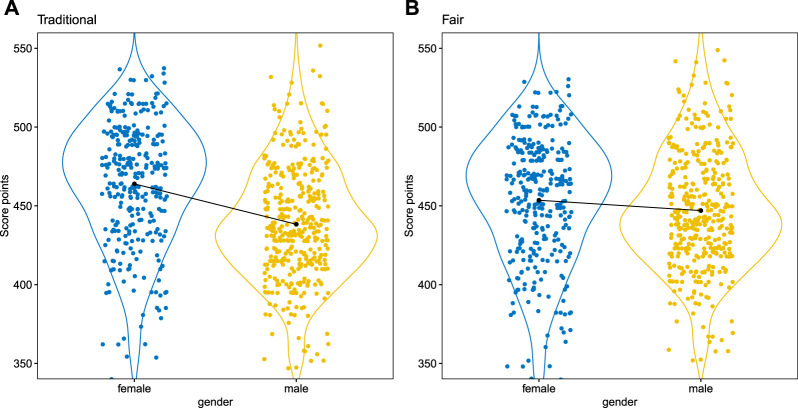
Protected attribute dependence plot of the traditional scorecard **(A)** vs. the fairness-corrected scorecard **(B)**. Note that the calibrated versions of the scores are used with 500 points at odds of 1/19 and 20 points to double the odds.

Figure 3 shows an example of the partial dependence profiles (cf. Section 3.2) for the traditional and the fairness-corrected model on one of the simulated data sets (Cramer’s V = 0.47, cf. Table 7). For these data no strong differences in performance are observed (0.554 vs. 0.560) but the GUI of 0.321 of the traditional model indicates unfairness. This is also reflected by the profile plots where a shift in the score point distributions can be noticed for the traditional model ( ${\bar{{Points}_{\;}}}_{female}=$ 463.96 vs. ${\bar{{Points}_{\;}}}_{male}=$ 438.32) in contrast to the fair model ( ${\bar{{Points}_{\;}}}_{female}=$ 453.57 vs. ${\bar{{Points}_{\;}}}_{male}=$ 447.01) while at the same time the standard deviation of the points for both models, which is often and indicator for the predictive power of the model, remains pretty similar: $\hat{\sigma }{\left(Points\right)}_{trad}=$ 39.28 vs. $\hat{\sigma }{\left(Points\right)}_{fair}=$ 38.68.

Along with these promising results another side effect can be noticed: As a consequence of gender-wise fairness correction there are different WOEs for both genders in all bins and consequently also different scorecard points for each gender as it can be seen in Table 8. Thus, the price of having a fair scoring model is different points with respect to the protected attributes (here: Gender). This can be difficult to explain to technically less familiar users or moreover, this can be even critical under the regulation constraints of the customers’ right to explanation of algorithmic decisions (Goodman and Flaxman, 2017). Not enough, a traditional plausibility check during the scorecard modeling process concerns monotonicity of the WOEs with respect to the default rates (Szepannek, 2020) which now has to be done for all levels of the protected attribute and is not necessarily given anymore after fairness correction. Note that also in our example the order of the default rates of the two bins with the highest risk of the variable Status has changed for the female customers.

**TABLE 8:** Comparison of the variable Status for the traditional model (left) and female (center) and male (right) gender in the fair model.

Bin	Dist	%Bad	Woe	Points	Dist|f	%Bad|f	Woe|f	Points|f	Dist|m	%Bad|m	Woe|m	Points|m
No checking account	0.281	0.452	0.723	−17	0.142	0.467	1.343	−34	0.397	0.447	0.527	−13
… < 0 DM	0.263	0.397	0.497	−12	0.145	0.522	1.117	−28	0.360	0.355	0.301	−8
0 < = … < 200 DM	0.063	0.205	−0.442	11	0.098	0.194	0.178	−4	0.034	0.231	−0.638	16
… > = 200 DM/salary	0.393	0.105	−1.222	29	0.615	0.103	-0.602	15	0.209	0.112	−1.418	36

Although in general the presented methodology can be applied to arbitrary machine learning models the changes in the data as induced by the fairness correction put even more emphasis on a deep understanding of the resulting model and corresponding methodology of interpretable machine learning to achieve this goal (cf. e.g., Bücker et al., 2021 for an overview in the credit risk scoring context). Further note that as it is demonstrated in Szepannek (2019) the obtained interpretations bear the risk to be misleading. For this reason other authors such as Rudin (2019) suggest restricting interpretable models and in summary a proper analysis of the benefits of using more complex models should be done in any specific situation (Szepannek, 2017).

For the simulations in this study only one protected attribute has been created which impacts only one of the predictor variables in a comparatively simple graph structure (Gender → Status → *Y*). For more complex data situations causal search algorithms can be used to identify potential causal relationships between the variables that are in line with the observed data (Hauser and Bühlmann, 2012; Kalisch et al., 2012). Then all descendants of the protected attributes must be corrected accordingly.

## 6 Summary

In this study, different definitions of fairness are presented from the credit risk scoring point of view as well as a fairness correction algorithm based on the concept of counterfactual fairness. Furthermore, the idea of population stability is transferred into a new group unfairness index which allows quantifying and comparing the degree of group fairness of different scoring models. In addition, partial dependence plots are proposed to visualize the fairness of a model with respect to some protected attribute. Based on these measures, a simulation study has been set up which makes use of a corrected version of the well-known German credit data. The results of the study are quite promising: Up to some degree fairness corrections are possible without strong loss in predictive accuracy as measured by the Gini coefficient on independent test data. Nonetheless, as inherent consequence traditional scores of fairness corrected models will typically differ with respect to protected attributes which may result in a new kind of problem under the perspective of the customer’s regulatory right for an explanation of algorithmic decisions. The explanation of algorithmic decisions gets even more complicated and future work has to be done in order to investigate the observed effects of our study for other classes of machine learning models such as random forests, gradient boosting, support vector machines, or neural networks.

## Data Availability

The original contributions presented in the study are included in the article/Supplementary Material; further inquiries can be directed to the corresponding author.

## Footnotes

1
https://www.lendingclub.com/.

2
https://community.fico.com/s/explainable-machine-learning-challenge.

3
https://archive.ics.uci.edu/ml/datasets/South+German+Credit+%28UPDATE%29

## Supplementary Material

Supplementary Table S1 Summary of the differences between the German credit data and the South German credit data.

## References

[B1] Agrawal A. Pfisterer F. Bischl B. Chen J. Sood S. Shah S. (2020). Debiasing Classifiers: Is Reality at Variance with Expectation? Available at: http://arxiv.org/abs/2011.02407 .

[B2] Bischl B. Kühn T. Szepannek G. (2016). “On Class Imbalance Correction for Classification Algorithms in Credit Scoring,” in Operations Research Proceedings 2014, Selected Papers of the Annual International Conference of the German Operations Research Society (GOR). Editors Lübbecke M. Koster A. Letmathe P. Madlener R. Peis B. Walther G., 37–43. 10.1007/978-3-319-28697-6∖_610.1007/978-3-319-28697-6_6

[B3] Bücker M. Szepannek G. Gosiewska A. Biecek P. (2021). Transparency, Auditability and eXplainability of Machine Learning Models in Credit Scoring. J. Oper. Res. Soc. in print. 10.1080/01605682.2021.1922098

[B4] Chouldechova A. (2016). Fair Prediction with Disparate Impact: A Study of Bias in Recidivism Prediction Instruments. Available at: http://arxiv.org/abs/1610.07524 . 10.1089/big.2016.0047 28632438

[B5] Cramér H. (1946). Mathematical Methods of Statistics. Princeton University Press.

[B6] Crook J. N. Edelman D. B. Thomas L. C. (2007). Recent Developments in Consumer Credit Risk Assessment. Eur. J. Oper. Res. 183, 1447–1465. 10.1016/j.ejor.2006.09.100

[B7] De Cnudde S. Moeyersoms J. Stankova M. Tobback E. Javaly V. Martens D. (2019). What Does Your Facebook Profile Reveal about Your Creditworthiness? Using Alternative Data for Microfinance. J. Oper. Res. Soc. 70, 353–363. 10.1080/01605682.2018.1434402

[B8] Dua D. Graff C. (2017). UCI Machine Learning Repository. Available at: https://archive.ics.uci.edu/ml/index.php .

[B9] EU Expert Group on AI (2019). Ethics Guidelines for Trustworthy AI.

[B10] European Banking Authority (2017). Guidelines on PD Estimation, LGD Estimation and the Treatment of Defaulted Exposures.

[B11] Friedman J. (2001). Greedy Function Approximation: A Gradient Boosting Machine. Ann. Stat. 29, 1189–1232. 10.1214/aos/1013203451

[B12] Goodman B. Flaxman S. (2017). European Union Regulations on Algorithmic Decision-Making and a "Right to Explanation". AIMag 38, 50–57. 10.1609/aimag.v38i3.2741

[B13] Groemping U. (2019). South German Credit Data: Correcting a Widely Used Data Set. Department II, Beuth University of Applied Sciences Berlin. Available at: http://www1.beuth-hochschule.de/FB/UnderScore/II/reports/Report-2019-004.pdf .

[B14] Hauser A. Bühlmann P. (2012). Characterization and Greedy Learning of Interventional Markov Equivalence Classes of Directed Acyclic Graphs. J. Machine Learn. Res. 13, 2409–2464. Available at: https://jmlr.org/papers/v13/hauser12a.html .

[B15] Henery R. J. Taylor C. C. (1992). “StatLog: An Evaluation of Machine Learning and Statistical Algorithms,” in Computational Statistic. Editors Dodge Y. Whittaker J. (Heidelberg: Physica), 157–162. 10.1007/978-3-662-26811-7_23

[B16] Hoffmann H. (1990). Die Anwendung des CART-Verfahren zur statistischen Bonitätsanalyse. Z. für Betriebswirtschaft 60, 941–962.

[B17] Kalisch M. Mächler M. Colombo D. Maathuis M. H. Bühlmann P. (2012). Causal Inference Using Graphical Models with the R Package Pcalg. J. Stat. Softw. 47, 1–26. 10.18637/jss.v047.i11

[B18] Kusner M. J. Loftus J. R. (2020). The Long Road to Fairer Algorithms. Nature 578, 34–36. 10.1038/d41586-020-00274-3 32020122

[B19] Kusner M. Loftus J. Russell C. Silva R. (2017). “Counterfactual Fairness,” in Proc. 31st int. Conf. Neural Information Processing Systems NIPS’17, Red Hook, NY, USA (Curran Associates Inc.), 4069–4079.

[B20] Lessmann S. Baesens B. Seow H.-V. Thomas L. C. (2015). Benchmarking State-Of-The-Art Classification Algorithms for Credit Scoring: An Update of Research. Eur. J. Oper. Res. 247, 124–136. 10.1016/j.ejor.2015.05.030

[B21] Louzada F. Ara A. Fernandes G. B. (2016). Classification Methods Applied to Credit Scoring: Systematic Review and Overall Comparison. Surv. Operations Res. Manage. Sci. 21, 117–134. 10.1016/j.sorms.2016.10.001

[B22] Lübke K. Gehrke M. Horst J. Szepannek G. (2020). Why We Should Teach Causal Inference: Examples in Linear Regression with Simulated Data. J. Stat. Educ. 28, 133–139. 10.1080/10691898.2020.1752859

[B23] O’Neil C. (2016). Weapons of Math Destruction: How Big Data Increases Inequality and Threatens Democracy. New York, NY, USA: Crown Publishing Group.

[B24] Pearl J. Glymour M. Jewell N. (2016). Causal Inference in Statistics – a Primer. Chichester, UK: Wiley.

[B25] Pearl J. (2019). The Seven Tools of Causal Inference, with Reflections on Machine Learning. Commun. ACM 62, 54–60. 10.1145/3241036

[B26] Robin X. Turck N. Hainard A. Tiberti N. Lisacek F. Sanchez J.-C. (2011). pROC: An Open-Source Package for R and S+ to Analyze and Compare ROC Curves. BMC Bioinformatics 12, 77. 10.1186/1471-2105-12-77 21414208 PMC3068975

[B27] Rudin C. (2019). Stop Explaining Black Box Machine Learning Models for High Stakes Decisions and Use Interpretable Models Instead. Nat. Mach Intell. 1, 206–215. 10.1038/s42256-019-0048-x PMC9122117 35603010

[B28] Siddiqi N. (2006). Credit Risk Scorecards: Developing and Implementing Intelligent Credit Scoring. Second edition. Wiley.

[B29] Szepannek G. (2020). An Overview on the Landscape of R Packages for Credit Scoring. Available at: http://arxiv.org/abs/2006.11835 .

[B30] Szepannek G. (2019). How Much Can We See? A Note on Quantifying Explainability of Machine Learning Models. Available at: http://arxiv.org/abs/1910.13376 .

[B31] Szepannek G. (2017). On the Practical Relevance of Modern Machine Learning Algorithms for Credit Scoring Applications. WIAS Rep. Ser. 29, 88–96. 10.20347/wias.report.29

[B32] Verbraken T. Bravo C. Weber R. Baesens B. (2014). Development and Application of Consumer Credit Scoring Models Using Profit-Based Classification Measures. Eur. J. Oper. Res. 238, 505–513. 10.1016/j.ejor.2014.04.001

[B33] Verma S. Rubin J. (2018). “Fairness Definitions Explained,” in Proc. Int. Workshop on software fairness FairWare ’18, New York, NY, USA. (ACM), 1–7. 10.1145/3194770.3194776

[B34] Xie S. (2020). Scorecard: Credit Risk Scorecard – R Package. version 0.3.1. Available at: https://CRAN.R-project.org/package=scorecard .

[B35] Youyou W. Kosinski M. Stillwell D. (2015). Computer-based Personality Judgments Are More Accurate Than Those Made by Humans. Proc. Natl. Acad. Sci. USA. 112, 1036–1040. 10.1073/pnas.1418680112 25583507 PMC4313801

