# Supplementary material for: Facing the Challenges of Developing Fair Risk Scoring Models
Source: Front Artif Intell. 2021 Oct 14;4:681915. doi: 10.3389/frai.2021.681915 (PMC8552888; doi:10.3389/frai.2021.681915)
Supplement: Supplementary file 1 [file Table1.pdf]

Supplementary Table S1 Summary of the differences between the German credit data and the South German credit data.

variable	level.new	level.old	good	bad
status	0<= ... < 200 DM	... >= 200 DM / salary for at least 1 year	0.07	0.05
status	no checking account	... < 100 DM	0.20	0.45
status	... < 0 DM	0 <= ... < 200 DM	0.23	0.35
status	... >= 200 DM / salary for at least 1 year	no checking account	0.50	0.15
credit_history	delay in paying off in the past	no credits taken/all credits paid back duly	0.02	0.08
credit_history	critical account/other credits elsewhere	all credits at this bank paid back duly	0.03	0.09
credit_history	existing credits paid back duly till now	delay in paying off in the past	0.09	0.09
credit_history	all credits at this bank paid back duly	critical account/other credits existing	0.35	0.17
credit_history	no credits taken/all credits paid back duly	existing credits paid back duly till now	0.52	0.56
savings	500 <= ... < 1000 DM	... >= 1000 DM	0.06	0.02
savings	100 <= ... < 500 DM	500 <= ... < 1000 DM	0.07	0.04
savings	... < 100 DM	100 <= ... < 500 DM	0.10	0.11
savings	... >= 1000 DM	unknown/no savings account	0.22	0.11
savings	unknown/no savings account	... < 100 DM	0.55	0.72
personal_status_sex	male : divorced/separated	male : divorced/separated	0.04	0.07
personal_status_sex	female : single	male : married/widowed	0.10	0.08
personal_status_sex	female : non-single or male : single	female : divorced/separated/married	0.29	0.36
personal_status_sex	male : married/widowed	male : single	0.57	0.49
property	real estate	unknown/no property	0.12	0.22
property	car or other	building society savings agreement/life insurance	0.23	0.24
property	unknown / no property	real estate	0.32	0.20
property	building soc. savings agr./life insurance	car or other	0.33	0.34
housing	own	for free	0.09	0.15
housing	for free	rent	0.16	0.23
housing	rent	own	0.75	0.62
foreign_worker	yes	no	0.05	0.01
foreign_worker	no	yes	0.95	0.99
installment_rate	>= 35	1	0.15	0.11
installment_rate	20 <= ... < 25	3	0.16	0.15
installment_rate	25 <= ... < 35	2	0.24	0.21
installment_rate	< 20	4	0.45	0.53
present_residence	< 1 yr	1	0.13	0.12
present_residence	4 <= ... < 7 yrs	3	0.15	0.14
present_residence	1 <= ... < 4 yrs	2	0.30	0.32
present_residence	>= 7 yrs	4	0.41	0.41
number_credits	>= 6	4	0.01	0.01
number_credits	4-5	3	0.03	0.02
number_credits	2-3	2	0.34	0.31
number_credits	1	1	0.62	0.67
people_liable	3 or more	2	0.16	0.15
people_liable	0 to 2	1	0.84	0.85
